# PROTOCOL: Protocol for a systematic review: Inter‐school collaborations for improving educational and social outcomes for children and young people

**DOI:** 10.1002/cl2.1011

**Published:** 2019-07-31

**Authors:** Paul Connolly, Jennifer Hanratty, Joanne Hughes, Christopher Chapman, Danielle Blaylock

**Affiliations:** ^1^ Campbell UK & Ireland, Centre for Evidence and Social Innovation Queen's University Belfast Belfast UK; ^2^ Centre for Shared Education Queen's University Belfast Belfast UK; ^3^ School of Education University of Glasgow Glasgow UK; ^4^ School of Psychology Queen's University Belfast

## 1 BACKGROUND

### 1.1 The problem, condition or issue

Over the last 20 years, inter‐school collaborations have become increasingly seen as a mechanism for improving educational and social outcomes amongst students. Countries around the world have incorporated a collaborative approach into their education systems in order to improve attainment, reduce inequality and address division along social, economic, religious or ethnic lines (Bell et al., 2006; Borooah & Knox, 2015; Chapman, Collins, Sammons, Armstrong, & Muijs, 2009; Duffy & Gallagher, 2014b).

Well known examples of inter‐school collaboration initiatives include, Beacon schools, which emerged in England and Wales in the late 1990s. Their aim was to ‘twin’ high performing schools with ‘failing’ schools and funding them to build partnerships to share best practice in order to improve performance (Rudd et al., 2000). More recently, the London Challenge and, later, its extension to other cities through the City Challenge built upon this approach by encouraging schools to work in partnership to raise standards and support the dissemination of best practice. Such initiatives have been replicated elsewhere, in Scotland for example with the School Improvement Partnership Programme (Chapman et al., 2014). Internationally, school networks have been developed in the USA, Europe and Hong Kong (Veugelers & O'Hair, 2005).

With regard to social outcomes, inter‐school collaborations have been particularly used to reduce prejudice and promote positive community relations in areas characterised by racial and ethnic divisions. In the context of divided societies education policy has consistently promoted ways to improve children's education and community cohesion through school‐level action (NCLB, 2001; DENI, 2009; Education and Inspections Act, 2006; Hansson, O'Connor Bones, & McCord, 2013). While ‘integrated’ or ‘desegregated’ education has been widely used to try to improve community cohesion, from Magnet schools in the USA to bi‐lingual, bi‐national schools in Israel where Arabs and Jews are educated together, inter‐school collaborations offer an alternative approach. Rather than drawing individuals from diverse backgrounds into a common school, inter‐school collaboration allows schools to retain their distinctive ethos while increasing meaningful and sustained contact between members of separate groups through educational activities (Hughes, Lolliot, Hewstone, Schmid, & Carlisle, 2012; Neins, Kerr, & Connolly, 2013). One of the most extensive examples of this approach is Northern Ireland where the Sharing Education Programme has, to date, supported more than 100 primary and post‐primary schools to engage in collaborative projects across religious and social divides and sought to improve both educational and social outcomes. This use of inter‐school collaborations to address the effects of racial and ethnic divisions and thus promote positive social outcomes has also been found in Israel (Berger, Abu‐Raiya, & Gelkopf, 2015), Bosnia‐Herzegovina (see Hansson et al., 2013 for discussion) and Cyprus (Zembylas, 2010a, 2010b) with varying levels of success.

There are many possible mechanisms through which school collaborations may bring about change in educational and/or social outcomes. Schools collaborating to share professional expertise may improve teaching practice and in turn lead to better educational outcomes for pupils. Schools collaborating to share resources, be they equipment, staff or learning resources may also improve the standards of teaching and educational outcomes. There are a number of theories that have been applied to explicate why collaboration may be a useful route to educational improvement (see Muijs, West, & Ainscow 2010 for details). For example, the COGNET programme (Greenberg, 1996) applied social constructivist theory to explain improvements in standardised test scores in intervention schools as compared to comparison schools. Collaborations for improving social outcomes tend to use contact theory as the main theoretical perspective, where increasing positive contact between different groups leads to improved social relations between the groups. Inter‐school collaborations can take many forms and have been variously named: federations, consortia, partnerships, networks, confederations and collegiate. These terms may be interchangeable to an extent but all refer to some form of schools working together towards a common goal. Various schemes for classifying and describing inter‐schools collaborations have been proposed in the literature. For example Hanford, Houck, Iler, & Morgan (1997) describe a four level typology of school collaborations that builds from exchanging information (networking), to joint activities (coordination), sharing resources (cooperation) and enhancing capacity of partners (collaboration). Atkinson, Springate, Johnson, & Halsey (2007) proposed three principle dimensions of inter‐school collaboration: organisational commitment and support; penetration of the collaboration; and holding a joint investment/vision. Others have classified inter‐school collaborations in terms of the different combinations of school types or partners involved. Chapman, Muijs, & MacAllister (2011) for example, described six different types of federation including cross‐phase federations involving schools of different phases (primary and secondary); performance federations focused on linking high and low performing schools; mainstreaming federations linking special and main stream schools; and academy federations or chains of schools with the same sponsor.

Evidence on the effectiveness of inter‐school collaborations has been inhibited by the variety of different approaches taken and also the difficulties in isolating the effects of inter‐school collaborations because of the complexity of initiatives, the lack of a control group, a lack of prospective evaluation and measurement of change over time. In relation to the London Challenge, for example, early indicators suggested that participating schools were experiencing significantly faster improvements in exam results than schools in the rest of England. The problem with seeing the London Challenge as a success story in inter‐school collaboration is that it involved much more than schools collaborating and happened at a time where other major changes were taking place. These included: increased funding; a push to improve data literacy in school leaders; greater support from Local Education Authorities and independent advisors who brokered tailored support for individual schools; and schools working together but not all to the same extent. Other changes that happened concurrently were the conversion of failing schools into academies (2002‐present) and the Teach First teacher training programme (2003‐present). The rapid improvements in London schools cannot be attributed solely to inter‐school collaboration because we cannot disentangle the effects of collaboration from the other supports provided to London Schools. In Northern Ireland, associations between children's involvement in shared education and positive improvements in attitudes and relationships have been found (Hughes, 2013). However, without a control group, it is difficult to establish whether changes are due to the collaboration itself or exogenous variables such as changes in government policy, changes in school leadership or wider political, social or community level changes that operate independently of school collaboration activities to improve outcomes.

Inter‐school collaborations have become increasingly prominent in education policy and practice as a mechanism for improving schools and educational and social outcomes. The problem is that many different models of school collaboration exist. It is not yet clear how effective school collaboration is and whether different models of collaboration are more or less effective than others.

### 1.2 The intervention

For the purposes of this systematic review, inter‐school collaboration will be defined as two or more schools working together on a sustained basis with the purpose of enhancing educational provision to improve educational and/or social outcomes for students. Types of collaboration can include: information sharing between teachers and/or students either face‐to‐face or virtually; teacher professional development and enhancement activities; the sharing of resources; and bringing students together for shared educational experiences.

As noted above, inter‐school collaborations can take many forms and are undertaken for a variety of reasons. The purpose of any collaboration will shape how that collaboration will be structured and managed, who will be involved and the outcomes targeted. For example, the purpose of a Beacon School was to act as a centre of excellence and their collaboration with other schools generally extended only to providing training or sharing best practice with staff from other schools (see Rudd et al. 2000). Federation of schools, often referred to as ‘hard federations’, will involve closer alignment of schools and may involve a single or shared management structure and sharing of resources (see Chapman, 2015). A final example are more informal arrangements where schools collaborate to deliver a specific curriculum or project and involves more direct contact between students (e.g., Neins, Kerr & Connolly et al 2013). Given the diversity of approaches to interschool collaborations we anticipate that detailed consideration of the goals, context and content of individual collaborations will be important in this review. Existing taxonomies will be used to guide classification of programmes for the purposes of synthesising findings (Atkinson et al., 2007; Muijs et al., 2010) but this will be an iterative process and classification will necessarily be flexible enough to accommodate any studies uncovered which do not fit with existing taxonomies.

To illustrate the type of study that would be included in this review, two studies that would be eligible for inclusion in our proposed review are briefly outlined below.

- Chapman et al. (2009) sought to identify the different ways federations operate and explore the impact of this variation in approach to changes in school performance. This quantitative study compared 264 schools grouped into 122 school federations with 264 matched comparator schools. Federation, in this study, encompassed a range of different approaches to collaboration, for example performance federations where high and low performing schools collaborate to raise standards in the underperforming school to cross‐phase federations where primary and secondary schools collaborate. National school level and pupil level data sets were used to compare performance of schools in the years prior to federation with performance after federation and compare this to matched comparison schools that were never federated.
- Kerr et al. (2011) evaluated the schools linking network project which aimed to reduce prejudice between groups of children based on religious, ethnic or cultural differences. This prospective evaluation compared 17 linked schools to a matched group of 27 schools who did not participant in the linking activities. Pupil attitudes, experiences and behaviour towards other groups was measured before and after participation in the school linking activities.

These two studies illustrate the diverse aims of studies that will be included in this review. Chapman et al. (2009) is an example of inter‐school collaboration where the main aim is to improve school performance and academic outcomes while in Kerr et al. (2011) school collaboration was motivated by a desire to improve cross‐community relations and social outcomes.

### 1.3 How the intervention might work

School collaboration is a complex process and one which is unlikely to automatically bring success without careful attention to context and content. Below we outline a range of possible ‘routes’ through which school collaboration may bring about positive change:

- Solving immediate problems: School collaborations may be precipitated by a need to solve immediate problems such as budget cuts. The economic benefits of school collaboration include economies of scale, reducing unnecessary duplication of facilities or maximising their use for little extra investment (Borooah & Knox, 2015).
- Raised attainment and expectations: Pairing underperforming schools with collaborators may raise standards and improve attainment through sharing best practice (Ofsted, 2011).
- Addressing vulnerable groups of learners: Pairing schools with a particularly good record of working with vulnerable students can prompt schools to rethink practice and expectations of vulnerable learners and have access to expertise in addressing particular student needs (Bell et al., 2006).

Widening opportunities, Interschool collaborations and sharing resources between schools can enable pupils to access a wider curriculum (Atkinson et al., 2007).

- Professional development: Teachers may benefit from professional development through sharing best practice and learning from expertise and subject specialists in their network (Bell et al., 2006).
- Social contact: The social benefits of increasing collaboration between schools are especially pertinent in the context of societies that are divided along religious or ethnic lines (Connolly, Purvis, & O'Grady, 2013; Duffy & Gallagher, 2014a, 2014b). Segregation in schools reduces opportunities for positive contact between separate groups. Examples from studies in Northern Ireland (Hughes et al., 2012; Neins et al., 2013), the USA (Hansson et al., 2013) and Bosnia‐Herzegovina (Hansson et al., 2013) have shown that establishing positive contact between groups in a school context can improve inter‐group relations.

Collaboration between schools comes with significant challenges including resistance from schools and communities, generating lasting and transferable effects, sustaining networks in the future and negotiating collaboration within a competitive education system. In addition to the routes through which collaboration may bring about positive outcomes, in‐depth case studies have highlighted some factors that may contribute to successful interschool collaboration. Factors associated with success according to (Atkinson et al., 2007) include: prior positive experience of collaboration, shared aims between collaborating schools; effective leadership; staff commitment; external support and funding.

As noted above interventions will vary in terms of their focus or purpose, the extent and depth of collaboration and who in the schools is actively involved. At the level of the individual child, the effect of inter‐school collaboration may vary according to the characteristics of the pupils involved. For example, in the case of school collaboration involving contact between pupils, not all young people will enter into collaboration with the same set of beliefs, attitudes, and experiences. Contact research suggests that some individuals and groups are more open to intergroup contact than others (Dixon, Durrheim, & Tredoux, 2005). For example, research suggests that the impact of intergroup contact may be weaker for members of minority status groups (Pettigrew & Tropp, 2013) and individuals from a more socially deprived background (Hughes, Blaylock, & Donnelly, 2015). Further, Turner and Cameron (2016) argue that young people who have a higher degree of confidence in contact will be more likely to positively engage in sustained intergroup relationships.

### 1.4 Why it is important to do the review

Previous reviews have provided very useful overviews of the area but no review has explicitly assessed the efficacy or effectiveness of inter‐school collaboration in controlled studies. Existing systematic reviews have been limited in scope and are now out of date. More recent reviews did not follow the rigorous process of a systematic review or were limited in their focus to UK based studies published in the last two decades.

There are currently four substantive reviews of the evidence regarding the effectiveness of inter‐school collaborations on student outcomes. Bell et al. (2006) undertook a rapid review that provided a map of existing studies and examined the impact of networks on students. A total of 19 studies met their inclusion criteria, with variation in study quality and outcomes. The review found that effective networks had clear goals, many involved partners such as higher education institutions, business and parents and all used shared expertise to improve outcomes for learners. Greatest improvements were seen when ‘at‐risk’ groups of pupils were targeted. The size and scale of a network had little impact; rather the quality of the collaboration was important. While this review represents a major effort in identifying the commonalities found in successful networks it was limited in its scope to studies published in English between 1995 and 2005 and involving three or more schools.

Atkinson et al. (2007) provided a narrative review of studies of school collaboration to summarise the different ways that schools work in partnership. This review focused on describing the nature and facilitators of inter‐school collaboration without an analysis of empirical evidence for the effectiveness of collaboration in improving outcomes. Atkinson et al. provide a very useful overview of the area and made progress in classifying different levels and forms of inter‐school collaboration, the potential benefits and factors which may influence successful collaboration. The review highlighted three main potential gains for schools collaborating: economic advantages of resource sharing; school improvement; and raising standards and forging relationships between schools. The review also highlighted the lack of good quality empirical evidence in the area at that time.

Dyson and Gallannaugh (2008) completed an EPPI‐Centre review that provided a scoping map of studies on school‐level actions to promote community cohesions. This extensive review was limited in scope to studies relating to the UK context published after 1987. This scoping map demonstrated that, at the time, much of the literature was descriptive with little focus on rigorous evaluation.

Armstrong (2015) recently provided another overview of the evidence on inter‐school collaboration in the UK only, published since 1999. While this review has identified a number of recent UK based studies it is largely based on qualitative evidence. The review did not conduct extensive searches and was restricted to only UK studies.

Given the limited scope of previous reviews, an up to date, systematic synthesis is now warranted.

Objectives

This systematic review will seek to answer the following key questions:

**Primary aim**

1. Do inter‐school collaborations improve educational and social outcomes for students?

**Secondary aims**

2. Do different types of inter‐school collaboration lead to different effects on educational and social outcomes for students?
3. For each type of inter‐school collaboration, is it possible to identify whether there are key characteristics that optimise their effectiveness on educational and social outcomes for students?
4. Do inter‐school collaborations have differing effects for students depending on their initial levels of attaining, their socio‐economic backgrounds, their gender, their ethnicity and/or their minority status? If so, do these differential effects vary in relation to differing types of inter‐school collaboration?

## 2 METHODOLOGY

### 2.1 Criteria for including and excluding studies

#### 2.1.1 Types of study designs

We anticipate that there will be few randomised (or cluster randomised) controlled trials and so we intend to include any controlled studies where intervention schools and students are compared to control or comparison schools and students either not participating in inter‐school collaborations or participating in alternative types of collaboration.

Studies with no control or comparison group, unmatched controls or national comparisons with no attempt to control for relevant covariates will not be included. Case studies, opinion pieces or editorials will not be included.

#### 2.1.2 Types of participants

Students of compulsory school age. This will vary from country to country but will typically cover students aged between 5–18 attending primary/elementary schools and secondary/middle/high schools.

The effects of school collaboration may differ depending on the characteristics of the participants involved (e.g. primary school or secondary education, pupils in special education, students from minority groups) and so pupil characteristics will be coded and, if sufficient studies are identified, examined as moderators.

#### 2.1.3 Types of interventions

Inter‐school collaboration will be defined as two or more schools, of any type, working together on a sustained basis with the purpose of enhancing educational provision to improve educational and/or social outcomes for students. Types of collaboration can include information sharing between teachers and/or students, either face‐to‐face or virtually; schools collaborating on teacher professional development and enhancement activities; the sharing of resources; or bringing students together for shared educational experiences.

Inter‐school collaborations need to involve sustained interaction between two or more schools. For the purpose of this review, 'sustained’ will be defined as occurring for at least one school term (a minimum of 10 weeks) and on a regular basis. One‐off or infrequent events, such as joint school trips, competitive events or sporting fixtures will therefore not be included. Initial teacher training activities involving multiple schools will also not be included because the focus is on teacher education and not collaboration per se.

Other collaborative approaches, such as professional learning communities, will not be included in this review, and have been reviewed elsewhere (Lomos, Hofman, & Bosker, 2011). We are interested in collaborations between schools as organisations, and not networks of individual teachers or professional development activities not driven by schools themselves.

The effectiveness of collaborations with external partners, such as businesses or community groups, is also beyond the scope of this review. Only school to school collaborations will be included.

Interventions whose sole aim is to reduce prejudice which do not also aim to deliver educational benefits will be excluded as a review on this topic is already underway (Keenan, Connolly, & Stevenson, 2015).

#### 2.1.4 Comparisons

We will include studies in which inter‐school collaborations are compared to ’no intervention’, ‘treatment as usual’, waitlist control or an alternative intervention (such as provision of additional resources to single schools not working in collaboration) or alternative type of collaboration. The type of comparison group (e.g., randomised groups versus matched comparison) will be carefully considered and if possible, examined as a moderator.

#### 2.1.5 Types of outcome measures

The primary outcomes of interest will be educational attainment and social outcomes. Educational outcomes include attainment in routine school tests and public examinations or standardised tests used for the purposes of an evaluation. Social outcomes will be: improvement in attitudes or reduction in prejudice towards students from a different socio‐economic or ethnic background; and/or increase in cross‐community friendships.

Secondary outcomes that will be considered will include any impacts on teachers (e.g., increased confidence, work‐based relationships) and on other, education‐related outcomes amongst students (e.g., attitudes towards school; school attendance; future career aspirations).

Adverse and unintended outcomes will also be included such as increasing fear, intergroup anxiety and resistance to future collaboration or reduced educational attainment.

Outcomes may be self, parent or teacher or researcher report or measured using standardised tests or measurement tools. In order to be included in any meta‐analysis, authors must have reported sufficient information to enable calculation of effect sizes and authors will be contacted to request this information if necessary.

### 2.2 Duration of follow‐up

Data will be extracted for all follow‐up periods reported in included studies.

### 2.3 Types of settings

We will only include interventions in primary or secondary schools or education settings (such as pupil referral units).

### 2.4 Search strategy

#### 2.4.1 Search limits

No date, location or language restrictions will be placed on the searches or included studies.

#### 2.4.2 Sources

We intend to search electronic databases for published and unpublished literature as well as extensive grey literature searches to identify unpublished reports. Finally we will consult a list of experts in the area for suggestions of additional unpublished/grey literature sources.

1. Electronic databases:
  a. British Education Index
  b. ERIC
  c. PsycInfo
  d. ProQuest Dissertation and Theses; UK & Ireland
  e. Educational Administration Abstracts (EBSCO)
  f. Australian Education Index
2. Research Registers and Websites
  a. Database of Abstracts of Reviews of Effectiveness
  b. National Technical Information Service https://ntrl.ntis.gov/NTRL/
  c. Evidence for Policy Practice Information and Coordinating Centre (EPPI‐Centre)
  d. Schools linking network (http://www.schoolslinking.org.uk/)
  e. Sharing Education Programme (http://www.schoolsworkingtogether.co.uk/)
3. Google & google scholar
  a. Search using key words (e.g., inter‐school collaboration, academy chain, school federation, school network, education network, school partnership, school cluster) and screen relevant articles on first two pages of google search results
4. Grey literature sources
  a. Open Grey (http://www.opengrey.eu/)
  b. National Foundation for Educational Research https://www.nfer.ac.uk/
  c. Digital Education Resource Archive (DERA) http://dera.ioe.ac.uk/
  d. Institute of Education Studies What works clearing house http://ies.ed.gov/ncee/wwc/publications_reviews.aspx
5. Conference abstracts and proceedings will be reviewed to identify potentially relevant studies. Conference searches will include:
  i. American Educational Research Association Repository (http://www.aera.net/Publications/OnlinePaperRepository/AERAOnlinePaperRepository/tabid/12720/Default.aspx indexing conference papers since 2010)
  ii. The Society for Research on Educational Effectiveness (https://www.sree.org/pages/conferences/index.php indexing conference papers since 2006)
6. Manual searches
  The latest issues of the top journals (in terms of included studies) will be manually checked towards the end of the retrieval process to ensure none of the most current evidence has been missed.
7. Expert Consultation
  Authors of prior studies and reviews will be contacted to obtain unpublished studies, studies in process and published studies missed in the database search.
8. Reference lists

The reference lists from prior reviews and included studies will be reviewed for potential studies. We will also conduct forward citation searching using Google Scholar to search for studies citing our included studies.

#### 2.4.3 Search terms

Given the nebulous way in which inter‐school collaborations are termed it is important that we pay careful attention to the search terms used to identify potentially relevant studies. In order to ensure a complete, sensitive and specific search strategy we intend to use a modified version of the Pearl Harvesting method (Sandieson, Kirkpatrick, Sandieson, & Zimmerman, 2010) to generate and refine our search terms. Briefly, this entails identifying relevant reviews and extracting their search terms. The terms generated from previous reviews will then be searched for in database thesauri in PsycInfo and the British Education Index and any new relevant terms added to the search string. The review authors will then assess the compiled list of search terms and add any terms that may be missing. We will compile separate term lists for participants (schools, pupils, school leadership etc.), intervention (e.g., collaboration, network, cooperative etc.) and study design (RCT, trial, control group, comparison group). Each search string will be reviewed and any redundant terms removed (e.g., ‘classroom’ removed in favour of ‘class*’).

To improve search specificity search terms are typically included for outcomes. For this review however we have decided not to include a search string for outcomes. This is because outcomes are often not clearly described in this literature so we do not want to risk reducing the searches sensitivity. The search strings will then be tested to ensure that key articles already identified (Berger et al., 2015; Chapman et al., 2009; Neins et al., 2013) are found.

See Appendix 1 for a detailed sample search strategy for PsycInfo (via OVID). Database‐specific variations to the search strategy (e.g., use of verified age limiters) will be used where possible to limit the number of irrelevant search results.

### 2.5 Description of methods used in primary research

To be included in this review studies must, at a minimum, compare intervention participants to a matched control group. We anticipate that most studies will assign participants to intervention and control groups at a class or school level. Where this is the case, clustering of pupils within classes and/or schools must be accounted for in the synthesis of results. We will check that studies have made adequate adjustments for clustering in estimates of intervention effects. If this was not done, we will request information on intra‐class correlations (ICCs) for individual studies directly from study authors in order to adjust the effect estimates and standard errors to account for clustering (Donner & Koval, 1980). If this is not available, we will use ICCs from an external source to correct for clustering as described in 16.3.4 of the Cochrane Handbook (Deeks, Higgins, Altman, & Green, 2011). ICCs from studies that provide the best match on outcome measures and types of clusters from existing databases (Ukoumunne, Gulliford, Chinn, Sterne, & Burney, 1999) or other studies within the review will be used.

The quality of non‐randomised controlled trials will be assessed using the Newcastle‐Ottawa scale (Wells et al., 2012) with some modifications to address appropriateness of analysis and allocation of participants to groups as suggested by Deeks et al. (2003). The quality of randomised controlled trials will be assessed using the Cochrane Risk of Bias tool (Deeks, Higgins, & Altman, 2011).

## 3 CRITERIA FOR DETERMINATION OF INDEPENDENT FINDINGS

### 3.1 Unit of analysis issues

The unit of analysis will be individual studies. Multiple‐reports of the same study will be used to extract all relevant data pertaining to the study but each study will only contribute one estimate of intervention effect per outcome. If studies report more than one estimate of an outcome (e.g., report two measures of attitude to school) we will retain only one measure of effect size. The measure with the best psychometric properties will be retained, or, if a measure is more commonly used across studies, we will retain the effect size for the more commonly used measure.

Where studies report outcome data adjusted for baseline differences between groups this adjusted outcome data will be preferred for synthesis. Where provided, change from baseline data will be preferred over post‐intervention outcome data.

Where follow‐up data is reported at multiple time points we will extract data for all follow up periods.

### 3.2 Studies with multiple treatment arms

In studies where two or more intervention groups are compared to a single control group the sample size of the control group will be halved to allow both intervention arms to be entered into meta‐analysis and avoid double counting of the control participants. This is assuming that both interventions are relevant inter‐school collaborations. If not then only data for the relevant intervention and control group will be included in analysis.

### 3.3 Details of study coding categories

All included studies will be coded using a coding instrument developed specifically for this review (see Appendix 2; Data Extraction Framework). The instrument will be piloted and refined before use. Data extraction/coding will be divided into characteristics of the participants, the intervention(s), comparison, outcomes and study design, details of the coding items for each of the PICOS is provided below. We will also extract bibliographic information, source descriptors, details of funding and relationships between the intervention evaluator and provider of the intervention.

Participants; number in each group, age, school setting, ethnicity, and relevant group membership (e.g., in the context of Northern Ireland identifying as catholic or protestant will be pertinent)

Intervention; the stated goal of the collaboration, who is involved, funding arrangements, management structure, duration, intensity of collaboration.

Comparison; Details of any comparison intervention and/or what the control group were exposed to.

Outcomes; All relevant data will be extracted in order to calculate effect sizes. If relevant information is not reported the information will be sought from study authors.

Study design: details of how participants were allocated to groups (including where applicable, details of randomisation procedures) in the case of experimental designs or in the case of observational controlled studies, details of how the control group was selected and any checks on baseline equivalence between intervention and control groups.

### 3.4 Screening and data collection

One reviewer (JH) will screen titles to remove obviously irrelevant results. The title and Abstract of the remaining records will then be ‘double screened’ independently by two reviewers. The full text of potentially relevant records will then be double screened independently by two reviewers. Any disagreements at this stage will be resolved by discussion, deferring to a third reviewer where necessary. To ensure reliability of coding two authors will independently code all of the included studies using a predefined data extraction form (see Appendix 2). Any disagreements will be discussed until a consensus is reached, involving the wider author team if necessary.

### 3.5 Statistical procedures and conventions

Separate analysis will be run per outcome and time period. We anticipate that tools used to measure outcomes will vary across studies and therefore plan to use standardised mean difference effect sizes for continuous outcome data and odds ratio for dichotomous data. Analysis of main effects and moderator analysis will be conducted for each outcome separately using RevMan version 5.3. Results of meta‐analyses will be presented visually using forest plots and described in the text. Random effects meta‐analysis will be used throughout because we expect there to be variation between studies in the interventions and populations studied. Moderator analysis will be conducted using meta‐regression (using STATA) where sufficient studies are available. Moderator analysis will attempt to identify characteristics of the intervention, participants and study design that are associated with smaller or larger effect size estimates. Intervention effects may vary as a function of both participant level characteristics (pupil age, ethnicity, minority group membership, gender) and intervention goals (e.g., collaborations aimed at improving educational attainment vs collaborations aimed solely at improving social outcomes.

The Q and I2 statistics will be used to assess the degree of heterogeneity in included studies. Sensitivity analysis will be used to explore reasons for heterogeneity.

Publication bias will be assessed through visual inspection of funnel plots and, where at least 10 eligible studies are found, Eggers regression test (Egger, Smith, Schneider, & Minder, 1997).

If there are insufficient studies to conduct meta‐analyses, we will provide a narrative synthesis of the studies, including a consideration of the overall quality (risk of bias) and quantity of evidence. This narrative synthesis will be organised by outcome.

## FUNDING INFORMATION

This review is being funded by a grant from The Atlantic Philanthropies, grant number 22676.

## CONFLICT OF INTERESTS

The Atlantic Philanthropies have provided significant support for the development of “shared education” in Northern Ireland, a particular model of inter‐school collaborations to improve educational outcomes and to promote better relationships between Catholic and Protestant communities. However, The Atlantic Philanthropies has no editorial control or influence over this proposed review.

Individual members of the review team have also undertaken evaluations of the effectiveness of different types of inter‐school collaborations that may be eligible for inclusion in this proposed review.

## AUTHOR CONTRIBUTIONS

Connolly will have overall responsibility for the design, conduct, analysis and write up of the systematic review. The team will have regular meetings to coordinate progress and ensure that all members contribute to all aspects of the review. However, and within this, the particular expertise and lead contributions of team members will be as follows: Content: C.C., J.H., D.B. Systematic review methods: P.C., J.H. Statistical analysis: P.C., J.H. Information retrieval (searching, screening and data extraction): J.H., D.B., P.C. Connolly and Hanratty both have substantial expertise and experience in the conduct of systematic reviews including Campbell reviews. Blaylock, Chapman and Hughes all have expertise in inter‐school collaboration.

## APPENDIX 1 Sample Search Strategy for PsycInfo

**Table:**

1	exp Nursery Schools/ or exp Technical Schools/ or exp High Schools/ or exp Schools/ or exp Junior High Schools/ or exp Graduate Schools/ or exp Nongraded Schools/ or exp Boarding Schools/ or exp Elementary Schools/ or exp Institutional Schools/ or exp Charter Schools/ or exp Military Schools/ or exp Middle Schools/
2	education/ or curriculum/ or elementary education/ or high school education/ or middle school education/ or multicultural education/ or nontraditional education/ or preschool education/ or private school education/ or public school education/ or religious education/ or remedial education/ or secondary education/ or special education/ or teacher education/
3	School*.ti,ab,id.
4	Education*.ti,ab.
5	curriculum.ti,ab.
6	1 or 2 or 3 or 4 or 5
7	((school* or educat*) adj2 cluster?).ti,ab.
8	((school* or educat*) adj2 chain).ti,ab.
9	((school* or educat*) adj2 collaborat*).ti,ab.
10	((school* or educat* or teach*) adj2 (collegiality or collegiate)).ti,ab.
11	((school* or educat*) adj2 (co?location or co?operat*)).ti,ab.
12	((school* or educat* or teach*) adj3 (confederation? or federation? or federated)).ti,ab.
13	((school* or educat* or teach*) adj3 network*).ti,ab.
14	(school* adj2 partner*).ti,ab.
15	(inter‐school* or inter?school*).mp.
16	((share or shared or sharing) adj2 (campus or curriculum or education or facilities or resources or services)).ti,ab.
17	joint curriculum.mp.
18	professional learning communit*.ti,ab.
19	learning organi?ation*.ti,ab.
20	Networked Learning Communit*.tw.
21	7 or 8 or 9 or 10 or 11 or 12 or 13 or 14 or 15 or 16 or 17 or 18 or 19 or 20
22	random*.tw.
23	(comparison or compare or comparing or compared).tw.
24	control group*.tw.
25	program evaluation/ or treatment effectiveness evaluation/
26	match*.tw.
27	pre‐post.tw.
28	quasi‐experiment*.tw.
29	effective schools research.mp.
30	22 or 23 or 24 or 25 or 26 or 27 or 28 or 29
31	6 and 21 and 30
32	"teaching school*".mp.
33	"Academy chain*".mp.
34	"Beacon* school*".mp.
35	"diversity pathfinders".mp.
36	"education action zone*".mp.
37	"excellence in cities".mp.
38	"city challenge*".mp.
39	"extended school*".mp.
40	"academy trust".mp.
41	"specialist school".mp.
42	"umbrella trust".mp.
43	"leading edge partner*".mp.
44	"education improvement partnership*".mp.
45	("area learning partner*" or "area learning communit*").mp.
46	31 or 32 or 33 or 34 or 35 or 36 or 37 or 38 or 39 or 40 or 41 or 42 or 43 or 44 or 45
47	6 and 21 and 30
48	47 or 46

## APPENDIX 2 Data Extraction Framework

**Table:**

**Study Eligibility**
Does the study involve collaboration between two or more schools?
Yes, No or Unclear
Does the study use a control group or comparison data to assess effect of collaboration? (I.e. comparing collaborating schools to schools not involved in collaboration?)
Yes, No or Unclear
Does the study set out to quantify the effect of school collaboration?
Yes, No or Unclear
**Reason for exclusion; e.g.**, No control or comparison schools, Only qualitative analysis, Not a school‐school collaboration (e.g., community‐school, family‐school, university‐school), Other ‐ make detailed notes
**General Info**
Reference Citation
Author contact details;
Study ID (to identify studies with multiple papers)
Report ID (to identify individual papers associated with a single study)
Year Publication:
Source: (journal, dissertation etc.)
Clinical Trial reference;
Study/report funded by?
Author affiliation to funder of the study?
Protocol information; (if available)
Other linked reports in the reference list?
Publication type (journal, dissertation, report, unpublished manuscript)
Where was the record found?
(E.g., named database, reference list, citation search of included studies, hand‐search, gov database, expert suggestion, author contact…)
**Study design**
Design; e.g., Case controlled, RCT, controlled observational, matched comparison
(NB if unsure give detail of how schools were recruited, grouped and compared)
Prospective or retrospective study?
(Did the study begin before schools began to collaborate or after the collaboration was already underway or completed?)
Type of analysis undertaken (e.g., intent‐to‐treat, available case analysis, multiple imputation)
**Context of collaboration**
Country
Type of collaboration (voluntary, national initiative, induced by incentives, forced etc.)
Leadership and governance structure
Drivers to collaboration (detail on why the collaboration was formed)
Scale of collaboration (does the collaboration encompasses all schools in an area or only some schools within it? include numbers of schools where possible)
Year collaboration began (important to know what policy context applied at the time of the study)
Year collaboration ended
Date study began
Date study ended
Who funded the Collaboration?
Is the author affiliated to the funder of the collaboration?
Context; extract any background/contextual information about the collaboration including any policy context, social context, etc.
School phases involved (Primary, secondary, post‐16, other, not‐stated, unclear)
School types (academy, controlled, grammar etc.)
Is there any other useful information about the context not covered in the questions above?

**Collaboration details**			
Name of program			
Stated aim of the collaboration			
Did the stated aim differ from the collaboration actually achieved?			
Who initiated that collaboration?			
Who is leading the collaboration?			
Who is involved		Governing body	
		Head teachers	
		Teachers	
		Pupils	
		Parents	
		Other (give details)	
Intervention target		Whole school improvement	
		Single subject	
		Social outcomes	
		Teacher professional development	
		Other	
Duration of collaboration		How long did the collaboration run?	
		Frequency of collaborative activities? (e.g., daily, weekly etc.)	
		Duration of collaborative activities	
		Group or individual? Number in group if applicable	
Organisation		Does the partnership have any formal, legal or statutory status?	
		Is governance or management of the schools shared?	
		What degree of organisational infrastructure supports the collaboration?	
		Do the schools have a common budget for collaborative activities?	
		Do the schools share any staff?	
Penetration		How many people within the school community are involved?	
		Do the activities cover a broad curricular base?	
		What is the expected longevity of collaboration?	
Joint investment/vision		Is there loss of independence?	
		Is there a strategic vision?	
		Is there shared responsibility and accountability for all outcomes?	
		Is there shared decision making?	
School resources (other than personnel) used in delivery		Funding (please give details)	
		Other (please specify)	
Non‐School resources (other than personnel) used in delivery		Funding (please give details)	
		Other (please specify)	
**Comparison**			
How was the control/comparison group constructed?			
Often little information is given but extract any info – it will be useful later for deciding what interventions/studies are comparable and can and cannot be combined in meta‐analysis.			
Were comparison schools matched in any way to control schools (e.g., schools matched on %free school meals, location, indices of deprivation, size?			
**Participants**			
Age (Mean, SD, Range)			
Any further detail on age? E.g. age at study entry or age at follow‐up? Is outcome data broken down by age group or age analysed as a mediator of intervention effect?			
Ethnicity			
Socioeconomic status (usually based on parents or local area stats);			
Parent information (e.g., single parent families, parent education)			
Gender			
School year(s);			
School type(s);			
School info (size, demographics, %FSM, etc.).			
Number of schools			
Number of participants			
Details of drop out/attrition at each stage and reasons for attrition			
Selection criteria: (how were schools/children recruited and any inclusion/exclusion criteria applied?)			
Withdrawals and exclusions			detail any participants who withdrew or were excluded and reasons given
Sample population			Describe the population from which the study sample was drawn
Sample size			
Location			
**Analysis**			
• Is the reported analysis adequate and correct?			
• Is the authors’ interpretation supported by the evidence?			
• Are there any biases/caveats raised or to be aware of?			
• Is there corroboration or triangulation of sources? (qual data)			
**Outcomes**			
List each outcome measured (in method section)			
Tool used to measure the outcome			
Extract any info on scale construction, what it purports to measure, references to scale manual or scale/measurement tool psychometric properties.			
Who completed each measure (child, teacher, parent, observer, other)			
Method of completion (interview, pen/paper, online etc.)			
Note format of data (continuous, dichotomous)			
Note any outcomes measured (according to trial protocol or method section that are not fully reported in the results).			
Timing of outcome measurement (for each outcome/scale used)			
Timing of outcome collection			
Extract all data presented for each outcome of interest			
Outcome	Results		
1)			
2)			
3)			
4)			
5)			
